# Accelerated Blood Clearance (ABC) Phenomenon Favors the Accumulation of Tartar Emetic in Pegylated Liposomes in BALB/c Mice Liver

**DOI:** 10.1155/2018/9076723

**Published:** 2018-01-16

**Authors:** Tamara C. M. Lopes, Débora F. Silva, Walyson C. Costa, Frédéric Frézard, José M. Barichello, Neila M. Silva-Barcellos, Wanderson G. de Lima, Simone A. Rezende

**Affiliations:** ^1^Laboratório de Pesquisas Clínicas, CIPHARMA, Escola de Farmácia, Universidade Federal de Ouro Preto, Ouro Preto, MG, Brazil; ^2^Laboratório de Nanotecnologia e Sistemas Nanoestruturados, Universidade Federal de Minas Gerais, Belo Horizonte, MG, Brazil; ^3^Laboratório de Tecnologia Farmacêutica, CCQFA, Universidade Federal de Pelotas, Capão do Leão, RS, Brazil; ^4^Laboratório de Farmacologia, CIPHARMA, Escola de Farmácia, Universidade Federal de Ouro Preto, Ouro Preto, MG, Brazil; ^5^Laboratório de Morfopatologia, Universidade Federal de Ouro Preto, Ouro Preto, MG, Brazil

## Abstract

Tartar emetic (TE) was the first drug used to treat leishmaniasis. However, its use was discontinued due to high toxicity. Association of TE with liposomes is a strategy to reduce its side effects. Pegylated liposomes (Lpeg) present lower rates of uptake by macrophages and prolonged circulation compared to their nonpegylated counterparts. However, repeated administration of Lpeg can cause an Accelerated Blood Clearance* (ABC)* phenomenon, whereby recognition of liposomes by antibodies results in faster phagocytosis. This work evaluated the effect of TE administration on histopathological aspects and the effect of the* ABC* phenomenon on targeting and toxicity in mice. Our results show that treatment with free or liposomal TE had no effect on the erythrocyte count, on liver and spleen weight, and on hepatic, splenic, and cardiac histology in mice. Severe lesions were observed on the kidneys of animals treated with a single dose of free TE. Treatment with TE in Lpeg after induction of* ABC* phenomenon caused a significant increase in Sb level in the liver without toxicity. Furthermore, mice treated with TE in liposomes showed normal renal histopathology. These results suggest site-specific targeting of Sb to the liver after induction of* ABC* phenomenon with no toxicity to other organs.

## 1. Introduction

Antimony has been used in therapeutics for centuries. Paracelsus introduced antimony as a general panacea in the 16th century and this substance was once praised as one of the Seven Wonders of the World. In 1905, Plimmer and Thompson showed the activity of sodium and potassium tartrate against trypanosomes [1]. The use of the tartar emetic (a trivalent antimonial) for the treatment of mucocutaneous leishmaniasis was first reported in 1913 by Gaspar Vianna [2] and the efficacy against visceral leishmaniasis (VL) was demonstrated by Di Cristina and Caronia in Sicily in 1915 [3], but later, this drug was found to be highly toxic [4]. Due to the serious side effects associated with its use, such as thrombocytopenia and electrocardiographic disturbance, tartar emetic was replaced by pentavalent antimonials (SbV) [5].

There is evidence that SbIII is substantially more potent than SbV [6]. This observation supports the hypothesis of an intramacrophagic metabolic conversion of SbV into SbIII, with SbIII being the toxic element to* Leishmania* in the intracellular state [7]. Trivalent antimony is considered a clastogenic agent; however, it is not mutagenic. Furthermore, antimony is not carcinogenic to humans [8]. In general, antimony accumulates in vascularized tissues and organs, primarily kidneys and liver, besides having a great affinity for spleen and blood components. A significant fraction of antimony in the blood of treated patients can be related to the huge affinity of SbIII for erythrocytes, due to the formation of complexes with organic compounds, such as glutathione (GSH) [1, 9].

The progress in liposome targeting systems applied to classical free drugs has proved successful for the treatment of diseases such as human leishmaniasis [10]. In the 1970s, the first results of a liposomal system aimed at leishmaniasis treatment were presented [11]. Alving and coworkers [12] reported the leishmanicidal activity of pentavalent antimonials encapsulated in liposomes in the treatment of hamsters infected with* Leishmania donovani* and demonstrated a reduction in the parasitic load 700-fold larger with the liposome formulation than with the free drug. When administered by the intravenous route conventional liposomes tend to be directed to organs such as the liver, spleen, and bone marrow, as they are phagocytized by cells of the mononuclear phagocytic system (MPS), which is also where the* Leishmania* parasite is located.

Pegylated liposomes are largely used as carriers in drug delivery systems in order to improve the efficacy of therapeutic agents. The presence of polyethylene glycol (PEG) on the surface of liposomes provides a steric barrier against opsonins that reduces the uptake of liposomes by the MPS cells [13, 14] and thus allows their uptake by other cells.

However, previous studies in rats and Rhesus monkeys showed that the administration of a first dose of blank pegylated liposomes stimulated an immune response that interfered with the pharmacokinetics and biodistribution of the second dose [15]. It was also observed that the plasma half-life of the second dose of pegylated liposomes dramatically decreased when given five days and up to four weeks after the first dose, due to a high uptake of liposomes coated with PEG by liver and spleen cells, referred to as the Accelerated Blood Clearance or ABC phenomenon. The mechanism for the induction of such a phenomenon was subsequently proposed by Ishida and coworkers [16]. The authors reported that anti-PEG IgM is produced by spleen cells in response to an injected dose of blank pegylated liposomes, which bind selectively to PEG present at the liposome surface, causing the activation of the complement system. Then, the opsonization of antibody-coated pegylated liposomes by the C3b fragment increases the uptake of this complex by macrophages.

Recently, Azevedo and coworkers [17] evaluated the effect of a pegylated liposomal formulation of meglumine antimoniate (MA) on the pharmacokinetics and distribution of Sb in SWISS mice and in dogs. The study showed, for the first time, the influence of liposome pegylation on the blood pharmacokinetics and distribution of Sb to the bone marrow of infected animals. It was shown that the treatment with a single dose of pegylated liposomes caused a significant improvement of Sb distribution to the bone marrow compared to Sb in conventional liposomes.

Considering that the* ABC* phenomenon can interfere with liposome targeting to macrophages, thus causing an increase in the concentration of SbIII in these cells and a decrease in its blood levels, this study is proposed to evaluate the impact of the* ABC* phenomenon on the targeting of Sb to the liver and the spleen. Furthermore, the effect of free and liposomal TE on the red blood cell count and on liver, spleen, heart, and kidney histology was evaluated.

## 2. Materials and Methods

### 2.1. Materials

Distearoylphosphatidylcholine (DSPC) and distearoylphosphatidylethanolamine-polyethylene glycol 2000 (DSPE-PEG) were obtained from Lipoid GmbH (Ludwigshafen, Germany); cholesterol (CHOL) was obtained from Dishman (The Netherlands BV) and tartar emetic (TE, C_8_H_4_K_2_O_12_Sb_2_·3H_2_O) was obtained from Sigma-Aldrich (Saint Louis, USA).

### 2.2. Preparation and Characterization of Liposomal Formulations

Two different liposomal formulations were prepared. The pegylated liposome (Lpeg) was made from DSPC, CHOL, and DSPE-PEG at molar ratios of 5 : 4 : 0.3 at 76.9 g/L. Conventional liposome (Lconv) was prepared using DSPC and CHOL (5 : 4), at 120 g/L. Liposomes were prepared using the freeze and thaw technique followed by extrusion, as described previously, with modifications [18–20]. Briefly, the lipids were solubilized in chloroform and the formation of the lipid film occurred by solvent exclusion using a rotary evaporator operating at 70 rpm at 60°C for 20 min under reduced pressure. For the preparation of blank liposomes, the lipid film was hydrated with phosphate-buffered saline (PBS), pH 7.2. The drug-containing liposomes were hydrated with an isosmotic TE solution at 80 g/L. Encapsulation of TE proceeded through 10 freeze and thaw cycles using liquid nitrogen and a water bath at 60°C. The liposomal preparations were extruded using a 0.2 *µ*m polycarbonate membrane (Isopore membrane filters, Millipore) at 250 psi/60°C to obtain unilamellar liposomes. These liposomes were then dialyzed for 24 h against PBS pH 7.2 by using a dialysis tubing cellulose membrane (Sigma-Aldrich, Saint Louis, USA).

The liposomes were characterized by mean particle size, polydispersity index, and zeta potential analyses through photon correlation spectroscopy coupled to electrophoresis on a Zetasizer instrument (Nano Series, Nano ZS, Malvern). The encapsulation efficiency was determined for the resulting liposome suspension after digestion of the sample with nitric acid, by electrothermal atomic absorption spectrometry (ETAAS) (Analyst AA600, Perkin Elmer, MA, USA).

### 2.3. Hemolytic Activity

The hemolytic activity was analyzed by incubating free TE (150 *μ*g/mL) or the pegylated and conventional liposomes containing TE (50 to 250 *μ*g/mL) or blank liposomes with a 5% human erythrocyte (O^+^) suspension for 1 h at 37°C. The erythrocyte suspension was centrifuged (1000 ×g for 10 min), and cell lysis was determined spectrophotometrically (540 nm) on an ELISA reader (Molecular Devices, Emax), as described by Löfgren and coworkers [21] and Valadares and coworkers [22]. The absence of hemolysis (blank control) or 100% hemolysis (positive control) was determined by replacing the liposomal formulation with an equal volume of PBS or distilled water, respectively. The results were determined by evaluating the percentage of hemolysis compared to the negative and positive controls.

### 2.4. Experimental Animals and Ethics Statement

Isogenic male BALB/c mice (6 to 8 weeks old) were maintained at the Centro de Ciência Animal of the Universidade Federal de Ouro Preto (CCA/UFOP) in cages containing four animals per group at a temperature between 21 and 25°C, under a 12 h dark and light cycle. Animals were given water and a commercial rodent diet (Nuvilab CR-II) ad libitum. The experimental protocol was approved by the Ethics Committee in Animal Experimentation from UFOP under the protocol number 2013/39.

### 2.5. Evaluation of the Toxicity of the Treatments in Healthy Animals

BALB/c mice were divided into eight experimental groups (*n* = 8) that were submitted to different treatments administered in two steps. In the first step, animals from groups one to five received PBS (200 *µ*L) via the tail vein and animals from groups six to eight were treated by the same route with blank pegylated liposomes, to induce the* ABC* phenomenon. The lipid dose was calculated according to Abu Lila and coworkers [23] in order to induce the production of IgM antibodies. Seven days after the first dose the animals received the following intravenously: (1) PBS; (2) free trivalent antimonial (SbIII) at 12 mg Sb/kg; (3) blank liposomes; (4) SbIII in conventional liposomes at 12 mg Sb/kg; (5) SbIII in pegylated liposomes at 12 mg Sb/kg; (6) SbIII in conventional liposomes at 12 mg Sb/kg; (7) blank pegylated liposomes; (8) SbIII in pegylated liposomes at 12 mg Sb/kg.

The animals were weighed and euthanized 14 days after the second treatment by an overdose of anesthetics (ketamine 10%, Syntec, 24.0 mg/Kg; xylazine, 2.3%, Sespo Indústria e Comércio/LTDA, 12.0 mg/Kg). After euthanasia, blood was collected via the abdominal artery and placed in EDTA tubes. Complete blood cell analysis was performed at the Laboratório de Imunopatologia (NUPEB, UFOP), by using an Auto Hematology Analyzer, BC-2800Vet. The heart, liver, spleen, and kidneys were collected for histopathological analysis. The total body mass and liver and spleen weight were determined.

### 2.6. Level of Antimony in the Spleen and Liver

In order to determine the concentration of antimony in the liver and spleen, the tissues were homogenized and digested with nitric acid in a dry block (MA 4004; Marconi, São Paulo, Brazil), as previously described by Schettini and coworkers [24]. Sb was quantified in the digested samples by ETAAS [24].

### 2.7. Histological Staining

Liver, spleen, kidney, and heart samples obtained from control and treated animals were fixed in 10% buffered formaldehyde solution, pH 7.4, for at least 48 h before histological processing. The samples were processed according to routine histological techniques and embedded in paraffin blocks. Sections were obtained with a 4 *µ*m thickness by using a microtome, followed by hematoxylin-eosin staining (Bio-Optica, Milano, Italy). Images were digitalized by using a Leica® DM5000 optical microscope through the Leica Application Suite software (version 2.4.1).

### 2.8. Statistical Analyses

Statistical analyses were performed using GraphPad Prism 5. Each set of results was first evaluated for normal distribution using Kolmogorov–Smirnov test. Normally distributed data were analyzed through one-way ANOVA followed by Tukey's test. For nonnormally distributed data a Kruskal–Wallis test followed by Dunn's tests was used. Differences were considered statistically significant where *p* < 0.05.

## 3. Results

### 3.1. Characterization of the Liposomes

Table 1 shows the results of liposome characterization including mean vesicle size, polydispersity index, and drug encapsulation efficiency. As observed, the liposome formulations showed similar mean vesicle sizes and a monodisperse size distribution (polydispersity index < 0.3) [25]. The average encapsulation efficiency of SbIII in Lconv and Lpeg was 13.5% and 14.0%, respectively.

### 3.2. Hemolytic Activity and Red Series Analysis

**Figure 1:**
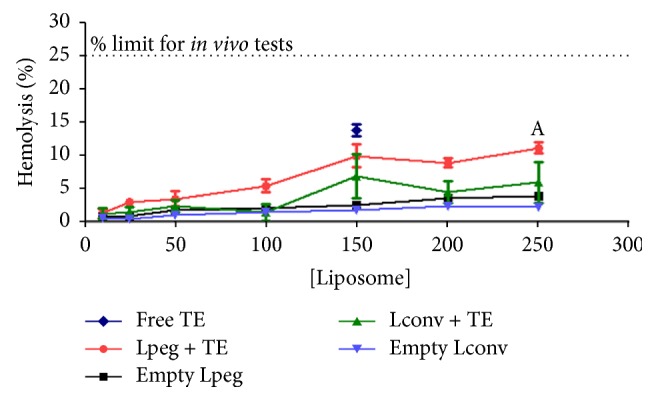
Hemolytic activity of the different formulations and free tartar emetic over a suspension of human O^+^ red blood cells (RBC), after 1 h incubation. Differences were considered statistically significant where *p* < 0.05 with respect to blank Lconv.

**Figure 2:**
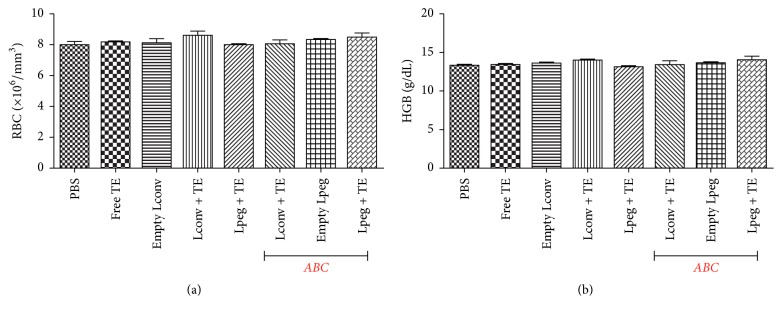
Hematological parameters in noninfected BALB/c mice after treatment. (a) represents erythrocytes [RBC] and (b) represents hemoglobin [HGB]. Results are represented as mean ± SD (*n* = 8).* ABC: Accelerated Blood Clearance* phenomenon.

Figure 1 shows the percentage of hemolytic activity. All treatments were weakly hemolytic (hemolysis < 15%) after incubation with different concentrations of the liposome formulations and of free TE.  Figure 2 shows the hematological parameters of erythrocytes [RBC] and hemoglobin [Hb] from mice treated with the different formulations and no differences were observed between the groups analyzed.

### 3.3. Biometric Analysis

**Figure 3:**
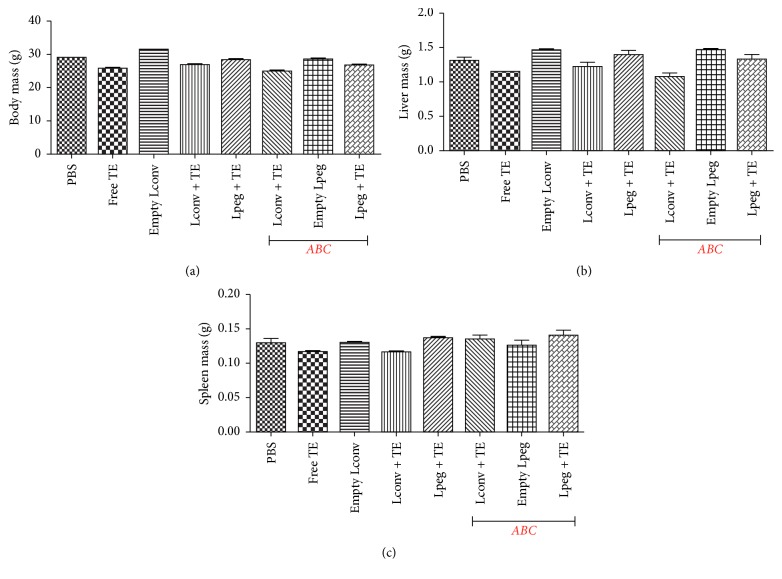
Biometric data of BALB/c mice. (a) represents the total body mass. (b) represents the liver mass, and (c) represents the spleen mass. Results are presented as mean ± SDs (*n* = 6).* ABC: Accelerated Blood Clearance* phenomenon.

No changes were observed in the body mass and in the liver and spleen weight from noninfected (healthy) mice subjected to different treatments (Figure 3).

### 3.4. Level of Antimony in the Spleen and Liver

**Figure 4:**
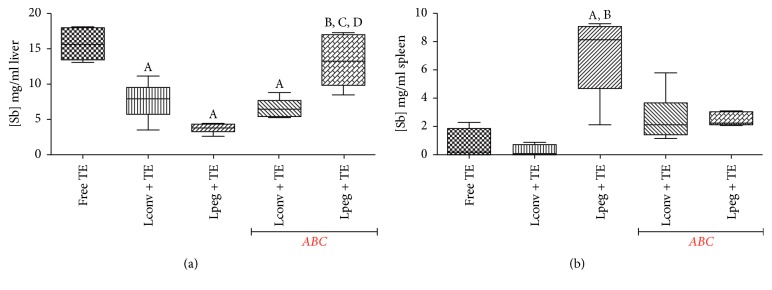
(a) Concentration of Sb in the liver of BALB/c mice. (b) Concentration of Sb in the spleen of BALB/c mice. The results are represented as median, minimum value, and maximum value (*n* = 4). Differences were considered statistically significant where *p* < 0.05 with respect to (A) free TE; (B) Lconv + TE; (C) Lpeg + TE; (D) Lconv + TE with* ABC* phenomenon.* ABC: Accelerated Blood Clearance* phenomenon.

Figure 4 shows the concentration of Sb determined in the liver and spleen 14 days after treatment. This analysis was performed to evaluate the concentration of Sb in mice submitted to different treatments, in the presence or absence of the* ABC phenomenon*. Interestingly, treatment with free TE produced the highest level of Sb in the liver. In the spleen, higher levels of Sb were observed in mice treated with TE in Lpeg without* ABC* phenomenon compared to the groups treated with free TE and TE in Lconv. Furthermore, treatment with TE in Lpeg after induction of the* ABC* phenomenon caused a significant increase in the level of Sb in the liver compared to treatment without* ABC* phenomenon. These results suggest that the induction of the* ABC* phenomenon can target TE in Lpeg to the liver in a site-specific fashion.

### 3.5. Histological Staining

Histopathological analyses of the hepatic tissues demonstrated that, in general, the animals subjected to the different treatments showed histological aspects similar to healthy tissues. The splenic architecture was preserved in all groups and no lesions were associated with the different treatments. Mice heart subjected to the different treatments showed normal architecture, without inflammation nor presence of tissue death areas.

**Figure 5:**
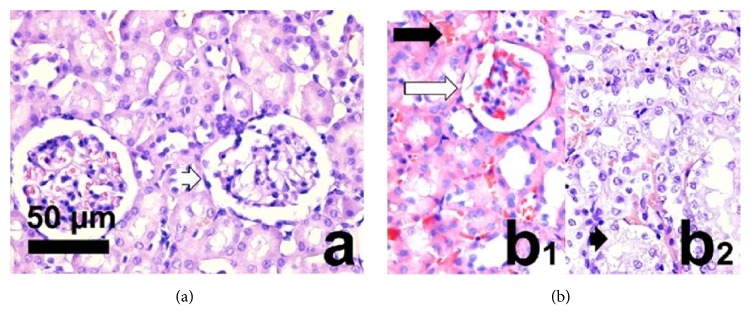
Photomicrographs of BALB/c mice kidney. (a) Normal histological micrograph with normal glomerulus (white arrowhead). (b_1_), (b_2_): Main lesions in BALB/c mice kidneys treated with free tartar emetic, corresponding to hyperemia (black arrow), glomerular sclerosis (white arrow), and tubular degeneration (black arrowhead). Images obtained with hematoxylin-eosin staining at a 440x magnification (bar = 50 *μ*m).

In the kidney, animals treated with 12 mg/kg of free TE showed different lesions such as hyperemia, tubular degeneration, hemorrhage, inflammation, and glomerular sclerosis. No other alterations were observed in the kidney of mice treated with the liposomal formulations of TE (Figures 5(a) and 5(b)).

## 4. Discussion

In this study we first prepared and characterized the liposomal formulations. The SbIII encapsulation efficiency in liposomes was similar to values reported in the literature (between 6.5 and 15.0%) [20, 26–28]. The vesicles showed a homogeneous size distribution. Extrusion is an advantageous approach with respect to the production of vesicles with a defined and well-characterized size distribution. This process is easy abd reproducible and does not introduce impurities into the vesicles [29]. The presence of negative charge on the surface of the vesicles affects the encapsulation efficiency. It was previously demonstrated that negatively charged liposomes are able to encapsulate larger amounts of antimony than positively charged liposomes [11].

Next, we tested the hemocompatibility of the liposomes through their ability to cause hemolysis. The* in vitro* effect of these formulations on red blood cells was examined, and we observed that the percentage of hemolytic activity was less than 15% for all the liposomal formulations and for free TE. According to Dobrovolskaia and coworkers [30], nanoparticles causing* in vitro* hemolysis between 1.0% and 25.0% can proceed to* in vivo* study. Subsequently, we demonstrated that the treatment with TE in liposomal formulations and free TE did not alter the number of red blood cells in BALB/c mice.

Reduction in the body and/or organs weight of animals is a simple and sensitive parameter for evaluating toxicity following exposure to harmful agents [31]. This work evaluated the body, liver, and spleen weight of treated mice and we observed that the formulations and free TE caused no alterations in these parameters. Dieter [32] also did not observe any changes in B6C3F1 mice bodyweight after intraperitoneal administration of trivalent antimonial at different doses (0, 6, 13, 25, 50, or 100 mg/kg) for 16 days.

The literature is scarce on the potential side effects caused by SbIII in the kidney; however, it is known that renal excretion constitutes the primary route for the elimination of antimonials [33]. In this work, we observed that the kidneys of animals treated with free TE by the endovenous route presented lesions such as hyperemia, tubular degeneration, hemorrhage, inflammation, and glomerular sclerosis. In contrast, mice treated with liposomal TE presented normal renal histology. Therefore, our work reinforces the proposed benefits of TE in Lpeg and TE in Lconv by demonstrating their reduced toxicity to the kidney tissues. De Melo and coworkers [26] showed that the encapsulation of a trivalent antimonial in pegylated liposomes reduced its acute toxicity and effectively delivered this drug to the parasite in experimental schistosomiasis. Castro and coworkers [20] showed that the intraperitoneal administration of 9 mg/kg of TE encapsulated in conventional liposomes was not toxic to the liver, heart, and kidney tissues of BALB/c mice infected with* L. infantum*.

Interestingly, in this study, we observed a greater amount of Sb in the liver when mice were treated with free TE in comparison with liposomal TE. The distribution of SbIII is determined by its great affinity for cells and after intravenous administration it is mainly distributed to the liver, kidney, and highly vascularized organs. Also, the organs rich in MPS cells show greater TE retention [34–36]. Collins et al. 1992 [37] observed a high concentration of Sb in the liver in comparison with the spleen and bone marrow after intravenous administration of sodium stibogluconate (SbV) in BALB/c mice infected with* L. donovani*.

In our study, an interesting result was observed in the liver of mice treated with conventional liposomal TE. The animals that received TE in Lpeg after induction of the ABC phenomenon exhibited high levels of Sb in the liver in comparison with the other groups, except for mice treated with free TE, which is an important observation considering that the liver is one of the targets of the parasite.

## 5. Conclusions

Our study showed that TE in liposomal formulations induced a low rate of* in vitro* hemolysis and had no effect on red blood cell count in mice. Furthermore, no alterations were detected in the body, liver, or spleen weight nor on the hepatic, splenic, cardiac, and kidney histopathology of mice treated with TE in liposomes. Treatment with TE in Lpeg after induction of the* ABC* phenomenon resulted in a significant increase in the level of Sb in mice liver. These results suggest site-specific targeting of antimony to the liver after induction of the* ABC* phenomenon with no toxicity to the organs evaluated.

## Figures and Tables

**Table 1:** Mean size, polydispersity index, zeta potential, and drug encapsulation efficiency. Results are represented as mean ± SD.

	Mean diameter (nm)	Polydispersity index	Zeta potential (mV)	Drug encapsulation efficiency (%)
*Lpeg + tartar emetic*	177.1 ± 0.9	0.07 ± 0.01	−2.59 ± 0.3	14%
Blank Lpeg	191.6 ± 1.6	0.06 ± 0.01	−4.87 ± 0.9	—
Lconv+ *tartar emetic*	197.3 ± 1.0	0.09 ± 0.03	−12.9 ± 1.7	13.5%
Blank Lconv	207.3 ± 0.4	0.09 ± 0.01	−10.2 ± 0.9	—
